# Comparative study on the clinical outcomes and prognosis of endovascular embolization and craniotomy clipping for the treatment of cerebral aneurysms

**DOI:** 10.12669/pjms.39.5.7401

**Published:** 2023

**Authors:** Gang Li, Shaojun Chen, Jing Han, Wanxi Pan, Ping Ji

**Affiliations:** 1 Gang Li, Department of Neurosurgery, The First People’s Hospital of Yichang, People’s Hospital of Three Gorges University, Yichang 443000, Hubei Province, P.R. China; 2 Shaojun Chen, Department of Neurosurgery, The First People’s Hospital of Yichang, People’s Hospital of Three Gorges University, Yichang 443000, Hubei Province, P.R. China; 3 Jing Han, Department of Neurosurgery, The First People’s Hospital of Yichang, People’s Hospital of Three Gorges University, Yichang 443000, Hubei Province, P.R. China; 4 Wanxi Pan, Department of Neurosurgery, The First People’s Hospital of Yichang, People’s Hospital of Three Gorges University, Yichang 443000, Hubei Province, P.R. China; 5 Ping Ji, The People’s Hospital of China Three Gorges University, The First People’s Hospital Of Yichang, Hubei Province, 443000, P.R. China

**Keywords:** Cerebral aneurysm, Endovascular embolization, Craniotomy clipping, Safety, Prognosis

## Abstract

**Objective:**

To investigate the safety and outcomes of endovascular embolization and craniotomy clipping in the treatment of cerebral aneurysms.

**Methods:**

We collected the clinical data of 106 patients with cerebral aneurysm who underwent surgical treatment (endovascular embolization, Group-A, n=55; craniotomy clipping, Group-B, n=51) in the First People’s Hospital of Yichang from January 2020 to May 2021. We compared surgical treatment indexes, treatment costs, neurological function before and after the treatment, incidence of postoperative complications and the prognosis after one-year follow-up between the two groups.

**Results:**

Endovascular embolization (Group-A) was associated with a shorter mean operation time and hospital stay, a lower mean intraoperative bleeding amount, and a higher mean treatment cost than craniotomy clipping (Group-B) (*P*<0.05). Compared with the pre-operative neurological function scores, the scores of both groups decreased after the surgery, and the mean post-operative score of Group-A was significantly lower than that of Group-B (*P*<0.05). Compared with Group-B , patients in Group-A had a lower overall complication rate (*P* < 0.05. Higher proportion of patients in Group-A had a good prognosis (*P* < 0.05).

**Conclusion:**

Endovascular embolization for the treatment of cerebral aneurysms is safe as it can shorten the operation time and hospital stay, reduce the incidence of neurological injury and complications, and have a favorable prognosis. However, the treatment is more expensive. Endovascular embolization can be selected for the treatment of cerebral aneurysms when economic conditions allow it.

## INTRODUCTION

Cerebral aneurysm, also known as intracranial aneurysm, is an abnormal focal dilation of the cerebral artery caused by a weakening of the inner muscular layer (intima) of the vessel wall.^1^,^2^ Thus, cerebral aneurysms are not tumors in a clinical sense.^3^ The causes of cerebral aneurysm remain unclear and may be related to congenital cerebral artery vascular malformations, hereditary conditions, infections, or trauma.^4^,^5^ Typical symptoms include unbearable headaches and hemifacial numbness, with a high mortality and disability rate. Therefore, early surgical treatment is important to promote the rehabilitation of patients and improve their prognosis.

Craniotomy clipping is one of the surgical procedures used to treat cerebral aneurysms. Its purpose is to block the blood supply of the aneurysm and prevent/avoid re-bleeding. However, it is a lengthy procedure that is associated with a significant trauma to the tissues with many postoperative complications,^6^ especially in elderly patients. Endovascular embolization procedure is minimally invasive and more easily accepted by patients physiologically and psychologically. Combined with imaging, endovascular embolization can effectively control bleeding from aneurysms and prevent their rupture.^7^ Since embolization is performed through the blood vessel, the procedure is less feared by the patients than craniotomy or nerve tractions necessary during other treatments. However, endovascular embolization has very strict technical requirements for the operating clinician, and the procedure is relatively difficult.^8^ While the efficacies of both surgical methods are well established, there is still no studies comparing the safety and post-operative prognosis of patients after the procedures.^9^,^10^ Therefore, the purpose of this study was to compare the safety and post-operative prognoses of endovascular embolization and craniotomy clipping for the treatment of patients with cerebral aneurysms.

## METHODS

We collected the records of 106 patients (43 men and 63 women) with cerebral aneurysms who underwent surgical treatment in our hospital from January 2020 to May 2021. Fifty-five patients were treated with endovascular embolization (Group-A) and 51 with craniotomy clipping (Group-B).

### Ethical approval

This study was approved by the medical ethics committee of the First People’s Hospital of Yichang (No. PJ-KY2022-16; July 13, 2022). The study was conducted in accordance with the Declaration of Helsinki.

### Inclusion criteria:

- Patients with cerebral aneurysms diagnosed based on cranial CT or digital subtraction angiography (DSA) imaging results.^11^
- Patients who underwent operation within 72 hours after the onset of symptoms and who tolerated the surgical procedure.
- All patients with normal bleeding and coagulation functions.
- Patients with complete clinical data.

### Exclusion criteria:

- Patients with Moyamoya disease, dissecting aneurysms or other vascular diseases.
- Patients with serious heart, liver, kidney or other organ diseases or those with serious infection.
- Patients had a history of cerebrovascular accidents such as a previous cerebral infarction.
- Patients with Alzheimer’s disease or other cognitive dysfunction diseases.
- Patients with malignant tumors.
- Pregnant and lactating women.

### Endovascular embolization

Patients underwent systemic heparinization two hours before the operation and were anesthetized by endotracheal intubation. The systolic blood pressure was maintained at 100 mmHg. Nimodipine (Chenxin Pharmaceutical, 50 mL:10 mg, Approval Number H20059048) was injected intravenously to prevent vasospasms, and angiography was performed through femoral artery puncture. Lidocaine carbonate (Shanghai Chaohui Pharmaceutical, 5 mL:0.1 G, Approval Number H31021072) was used for infiltration anesthesia at the groin femoral artery to fix the blood vessels and prevent vasospasms. The surgeon made an incision for femoral artery puncture to determine the location, size, and shape of the cerebral aneurysm.

Under the guidance of digital subtraction angiography (DSA), the surgeon punctured the 6F arterial sheath and sent it to the internal carotid artery and vertebral artery under Seldinger technology. Next, the microcatheter was inserted in the middle of the cerebral aneurysm cavity through the microcatheter, and the appropriate spring ring was selected according to the size of the cerebral aneurysm. For large aneurysms, the surgeon used a stent to embolize it until the angiographic cerebral aneurysm disappeared, then the catheter was pulled out, and the incision was sutured and pressurized. After the operation, 5000 U of low molecular weight heparin calcium injection (Hebei Changshan Biochemical Pharmaceutical, specification: 0.4 mL, Approval Number H20063910) were administered subcutaneously every 12 hours.

### Craniotomy clipping

After the anesthesia, Nimodipine (Chenxin Pharmaceutical, 50 mL:10 mg, Approval Number H20059048) was injected intravenously to prevent vasospasms, and the cerebral aneurysm was located based on the imaging diagnosis. The surgical approach was to select the perianal point of the lateral fissure, make an arc incision on the patient’s head under the microscope to drain the cerebrospinal fluid, cut the scalp, peel off the tissue, and expose the skull. After drilling the skull, the surgeon used a milling cutter to make a bone window; after opening the dura mater, the aneurysm was exposed backward along the internal carotid artery to determine the parent artery and the neck of the aneurysm.

Next, the surgeon used a temporary aneurysm clamp to block the parent artery according to the size of the aneurysm. After clamping the aneurysm and checking its condition and verifying that bleeding had stopped, the surgeon sutured the meninges and scalp incision and placed drainage under the skin. Nimodipine and other anti-infective drugs were administered for prevention and treatment after the operation. Usually, the drainage tube was pulled out two days after the operation when appropriate.

### Basic clinical data and the following relevant indexes were collected before and after the operation:

1. Perioperative indicators and treatment costs;
2. Neurological function status before and after the operation as measured by the National Institutes of Health Stroke Scale (NIHSS)^12^ with a total score of 42 (the higher the score, the more severe the neurological injury);
3. Incidence of complications, including hydrocephalus, intracranial infection, vasospasm, and cerebral infarction;
4. The Glasgow Coma Score^13^ to divide the patients according to their prognosis into five grades (Grade 1=death, Grade 2=vegetative survival, Grade 3=severe disability, Grade 4=mild disability, and Grade 5= favorable prognosis).

### Statistical analysis

We used SPSS 24.0 for the statistical analysis and considered differences with *P*<0.05 as statistically significant. The measurement data conforming to the normal distribution are represented by means and standard deviations (*χ̅*±*S*) and were compared using a t-test; count data [n (%)] were used for comparisons using *χ^2^* test.

## RESULTS

A total of 106 patients (43 men and 63 women) met the inclusion criteria, with the ages ranging from 38 to 81 years, with an average of 58.83±10.23 years. Group-A had 55 patients who underwent endovascular embolization, and Group-B included 51 patients who underwent craniotomy clipping. There were no significant differences in the basic data between the two groups (*P*>0.05), as shown in Table-I.

**Table-I:** Comparison of basic data between the two groups [n (%),*χ̅*±*S*].

Group	n	Sex	Age (years)	Aneurysm location		
	
		Men/Women		Anterior cerebral traffic	Posterior cerebral traffic	Middle artery
Group-A	55	19/36	59.52±10.18	12 (21.8)	9 (16.4)	34 (61.8)
Group-B	51	24/27	58.07±10.34	12 (23.5)	11 (21.6)	28 (54.9)
*χ^2^*/*t*		1.719	0.726	0.631		
*P*		0.190	0.469	0.730		

Compared with Group-B, patients in Group-A had shorter operation times and hospital stays. Endovascular embolization was associated with less intraoperative bleeding amounts, and higher treatment costs (*P*<0.05), as shown in Table-II. The postoperative neurological function scores of patients in both groups decreased after the operation, and the mean score in Group-A was significantly lower than that in Group-B (*P*<0.05) (Table-III).

**Table-II:** Comparison of treatment indexes and costs between the two groups (*χ̅*±*S*).

Group	n	Operation time (minutes)	Length of stay (days)	Intraoperative bleeding volume (mL)	Treatment cost (thousand yuan)
Group-A	55	75.89±11.65	12.14±2.22	83.81±14.21	119.77±15.98
Group-B	51	109.41±4.27	16.56±1.67	110.76±11.37	66.28±9.74
*t*		13.184	11.618	10.813	20.967
*P*		<0.001	<0.001	<0.001	<0.001

**Table-III:** Comparison of NIHSS neurological function scores between the two groups before and after the operation (*χ̅*±*S*).

Group	n	Preoperative	After operation
Group-A	55	15.76±3.09	6.71±2.42*
Group-B	51	15.45±3.25	10.88±2.88*
*t*		0.507	8.088
*P*		0.613	<0.001

***Note:***

* refers to the comparison with the pre-operative score, *P*<0.05.

The total incidence of complications in Group-A was significantly lower than that in Group-B (P<0.05) (Table-IV). Compared with Group-B, Group-A had a higher proportion of favorable prognoses and a lower proportion of poor prognoses (*P*<0.05; Table-V).

**Table-IV:** Incidence of complications in the two groups [n(%)].

Group	n	Hydrocephalus	Infection	Vasospasm	Cerebral infarction	Total incidence
Group-A	55	2 (3.6)	5 (9.1)	2 (3.6)	0	9 (16.4)
Group-B	51	5 (9.8)	9 (17.6)	6 (11.8)	2 (3.9)	22 (43.1)
*χ^2^*		-	-	-	-	10.145
*P*		-	-	-	-	0.038

**Table-V:** Comparison of prognoses between the two groups [n (%)].

Group	n	Favorable prognosis	Poor prognosis			

		Good recovery	Mild disability	Severe disability	Vegetative survival	Death
Group-A	55	41 (74.5)	9 (16.4)	3 (5.5)	1 (1.8)	1 (1.8)
Group-B	51	28 (54.9)	12 (23.5)	5 (9.8)	4 (7.9)	2 (3.9)
χ^2^		4.494				
P		0.034				

## DISCUSSION

We compared the clinical outcomes of endovascular embolization and craniotomy clipping in the treatment of patients with cerebral aneurysms. In theory, endovascular embolization produces less trauma and is highly precise, while craniotomy clipping produces more collateral trauma and is risky. Our results showed that clinical outcomes of patients treated using endovascular embolization were significantly better than those treated by craniotomy clipping.

Zhou D et al.^14^ compared the therapeutic outcomes of craniotomy clipping and endovascular embolization in patients with subarachnoid hemorrhage, and showed that the operation time and hospital stay of patients that underwent endovascular embolization were significantly shorter than those of patients that received craniotomy clipping. In addition, study by Zhao J et al.^15^ showed that endovascular embolization treatment can lower the impact on immune function, reduce adverse reactions, shorten hospital stay, and fully improve the curative effects. Unfortunately, we found that the average treatment cost of endovascular embolization is nearly twice that of craniotomy clipping. Earlier studies by Manabe H et al.^16^ had shown that the cost of craniotomy clipping was significantly lower than that of endovascular embolization. Thus, these two treatment methods have their own advantages and disadvantages. While craniotomy clipping is associated with substantial trauma to tissues and causes stress and fear in patients, the procedure can cure and completely ligate cerebral aneurysms at a relatively low cost. Therefore, craniotomy clipping may be an appropriate treatment when cost is a consideration.^17^ Endovascular embolization, on the other hand, is a minimally invasive treatment with short operation duration and little intraoperative bleeding. However, it is associated with a high cost. Endovascular embolization should be a method of choice when affordable as it provides superior rehabilitation and prognosis.^18^

The results of our study showed that the postoperative neurological function scores in Group-A were significantly lower than those in Group-B, indicating that endovascular embolization results in less nerve damage in patients with cerebral aneurysms. Hwang JS et al.^19^ reported that the incidence of neurological and cardiac complications after craniotomy clipping was higher than that after endovascular embolization. Endovascular embolization uses a microcatheter to implant a coil into the cerebral aneurysm through the femoral artery to block the blood supply of the aneurysm without craniotomy. The procedure is done without pulling nerves, thereby avoiding nerve injury and facilitating the recovery of patients’ nerve function.^20^,^21^ By contrast, craniotomy clipping requires opening of the skull, which can lead to nerve damage during the operation. Endovascular embolization, thus, is superior to craniotomy clipping for preserving neurological function of the patient during the procedure.

Our results showed that the total incidence of complications in Group-A was lower than that in Group-B, suggesting that endovascular embolization has better clinical outcomes than craniotomy clipping. Hydrocephalus infections, vasospasms and cerebral infarctions are common complications after a cerebral aneurysm surgery. The prognosis of patients is closely related to the occurrence of complications.^22^ Liu Q et al.^23^ developed a biodegradable magnesium titanium alloy biological stent and used it during an endovascular embolization. They established a hemodynamic model using a three-dimensional (3D) computer hemodynamic numerical simulation technology. The results of their study showed that endovascular embolization had better effects than craniotomy clipping in the treatment of intracranial aneurysms as their system provided a lower risk of complications and postoperative mortality and a stable vascular blood flow.

The lack of data on the hemodynamic characteristics of our patients are another limitation of our study. Araya et al.^24^ have reported that the decrease in cerebrospinal fluid volume through the craniotomy clipping provides an advantage by reducing brain edema. However, we found that the incidence of brain edema in Group-B was higher than that in Group-A. It is possible that the hemostatic materials and electric cautery interference used during craniotomies led to postoperative adhesions, which increased the risk of brain edema.

Our results showed that the prognosis of patients with aneurysms were better after endovascular embolization than after craniotomy. Zou et al.^25^ also showed that the survival of patients treated with endovascular embolization was higher than that of patients treated with craniotomy, which is consistent with our results. However, according to Zhang et al.^26^, aneurysms are more likely to recur after endovascular embolization than after craniotomy clipping. Craniotomy clippings using a microsurgical clipping technology are less traumatic than traditional craniotomies. Therefore, the aneurysm recurrence after endovascular embolization or craniotomy clipping needs to be further studied.

### Limitations

This study was conducted at a single center using retrospective data, and had a small sample size. Moreover, additional postoperative treatments that may have contributed to the prognosis of patients, were not studied. Additionally, as we aimed to focus on cerebral complications in this study, other noncerebral complications were not studied, and they should be further investigated in the future research.

## CONCLUSION

Patients with cerebral aneurysms may choose an endovascular embolization treatment when economic conditions permit it as the procedure seems to have better clinical outcome and favorable prognosis than craniotomy clipping, and it can reduce the neurological injury of patients.

### Authors’ contributions:

**GL** conceived and designed the study. **SC, JH, WP and PJ** collected the data and performed the analysis. **GL** was involved in the writing of the manuscript and is responsible for the integrity of the study. All authors have read and approved the final manuscript.
